# Severe Hypertrophic Cardiomyopathy Associated With Hydroxychloroquine in a Young Woman With Systemic Lupus Erythematosus: A Case Report and Review of the Literature

**DOI:** 10.7759/cureus.61452

**Published:** 2024-05-31

**Authors:** Bernard Brown, Christy Joseph, Varsha Talanki, Adam S Budzikowski, Samy I. McFarlane, Sabu John

**Affiliations:** 1 Internal Medicine, Downstate-Health Science University, Brooklyn, USA; 2 Cardiology, Downstate-Health Science University, Brooklyn, USA; 3 Cardiovascular Medicine – Electrophysiology (EP) Section, Downstate-Health Science University, Brooklyn, USA

**Keywords:** systemic lupus erythematosus, cardiotoxic agents, antiphospholipid antibody syndrome (aps), non obstructive hypertrophic cardiomyopathy, libman sacks endocarditis (lse), hydroxychloroquine (hcq), drug-induced cardiomyopathy, cardiomyopathy

## Abstract

The use of the antimalarial drug hydroxychloroquine is a standard treatment in patients with systemic lupus erythematosus. It helps reduce disease-associated damage, prevents disease flare, and improves overall survival. The mechanism of action of hydroxychloroquine includes interference with lysosomal degradation of cells leading to the accumulation of vacuoles. Retinopathy is a well-described adverse effect of hydroxychloroquine, thus requiring screening with an ophthalmologist after prolonged use. Although rarely reported, cardiac adverse effects of hydroxychloroquine can also occur. In this report, we present a case of a 23-year-old woman with systemic lupus erythematosus on hydroxychloroquine who presented with stroke possibly due to Libman-Sacks endocarditis and was found to have severe hypertrophic cardiomyopathy on transthoracic echocardiogram.

## Introduction

Hydroxychloroquine is a recommended treatment for systemic lupus erythematosus at any stage and it is given in doses of 5 mg/kg [1]. This leads to a decrease in lupus flares and helps prevent lupus nephritis [2]. Its mechanism of action involves down-regulating toll-like receptor activity [3]. This, in turn, leads to the inhibition of lysosomal enzyme activity, which results in the buildup of vacuoles containing lipids and glycogen. One side effect of hydroxychloroquine is retinal toxicity, requiring eye screening with an ophthalmologist after prolonged use [1]. Another rare side effect is reversible hypertrophic cardiomyopathy, which can be explained by the buildup of intercellular vacuoles [3].

## Case presentation

A 23-year-old woman with systemic lupus erythematosus (SLE) since the age of 14 complicated by lupus nephritis class V and a history of lupus serositis treated with pericardiocentesis had multiple episodes of venous thromboembolism. She also had hypertension and presented with right-sided weakness and aphasia. She was last known well more than 12 hours prior to admission. Her home medications included hydroxychloroquine, colchicine, mycophenolate, apixaban, losartan, nifedipine, and labetalol. Her vital signs on presentation included a temperature of 98 °F, blood pressure of 141/119 mmHg, heart rate of 105 beats/minute, and respiratory rate of 19 breaths/minute. Her physical exam was significant for right upper and lower hemiparesis, right facial droop, and aphasia.

**Figure 1:**
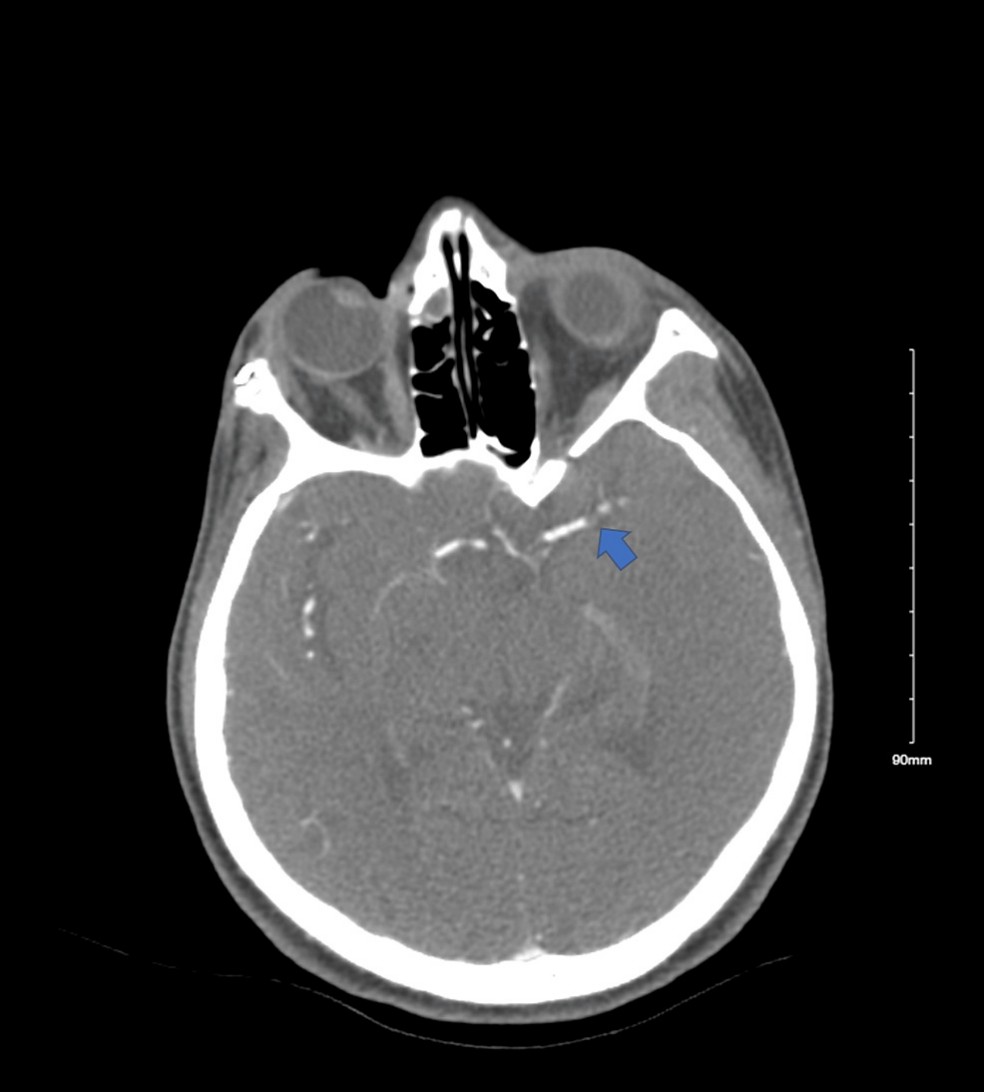
A computed tomography angiogram depicting an occlusion in the M1 region of the middle cerebral artery (blue arrow)

**Figure 2:**
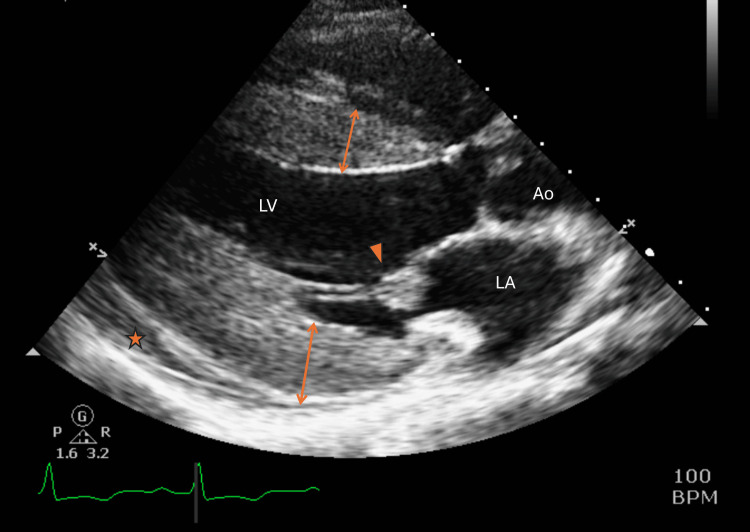
Transthoracic echocardiogram depicting increased posterior wall (1.8 cm), intraventricular septum thickness (1.5 cm) (orange double arrow) at end-diastolic, pericardial effusion (orange star), and Libman-Sacks endocarditis (orange arrowhead) LV: left ventricular, LA: left atrium, Ao: aorta

**Figure 3:**
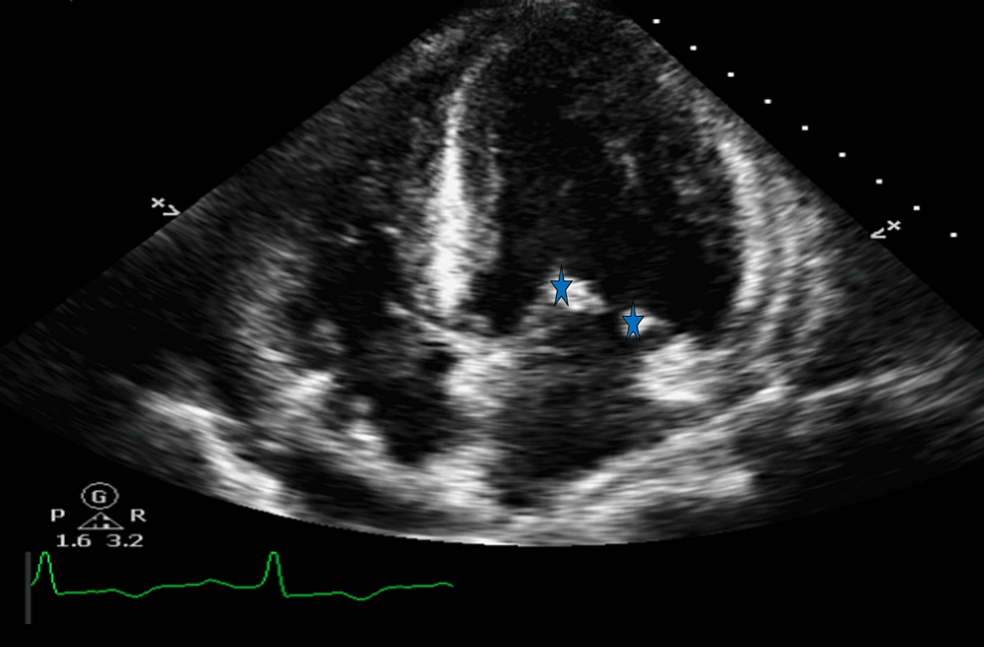
Transthoracic echocardiogram in the four-chamber view depicted Libman-Sacks endocarditis on the mitral valve (blue stars)

The patient’s laboratory findings included sodium 136 mmol/L, potassium 4.5 mmol/L, chloride 102 mmol/L, bicarbonate 19 mmol/L, blood urea nitrogen (BUN) 10 mg/dL, creatinine 1 mg/dL, glucose 91 mg/dL, white blood cells 5.1 K/uL, hemoglobin 6.7 g/dL, mean corpuscular volume (MCV) 67.1 fL, and platelets 255 K/uL. A computed tomographic angiography of the brain showed left M1 middle cerebral artery occlusion (Figure 1). The bilateral lower extremity Doppler was negative for acute thrombosis. As part of the stroke workup, a transthoracic echocardiogram (TTE) was done, which showed a left ventricular ejection fraction of 65%, severe left ventricular hypertrophy, moderate pericardial effusion, and moderately thickened mitral valve (MV) leaflets (Figures 2, 3). She was admitted to the Neurology intensive care unit for stroke management and was started on aspirin, atorvastatin, and a heparin drip. The patient did not receive alteplase for being out of the time window and having an ASPECTS (Alberta stroke program early CT score) of 6 was unfavorable for thrombectomy. The rheumatology service was consulted for the medical management of lupus in the setting of recurrent thrombosis. It was recommended to restart her home hydroxychloroquine and to obtain a laboratory workup for lupus and antiphospholipid syndrome. There were positive test results for dsDNA, beta 2 glycoprotein, cardiolipin antibodies, and lupus anticoagulant antibodies. The patient was diagnosed with antiphospholipid syndrome. She was then started on low molecular weight heparin, with an eventual transition to warfarin when her INR reached the goal of 2-3. A transesophageal echocardiogram with a bubble study that was done for further evaluation for a stroke causing emboli showed no thrombus in the left atrial appendage and severely thickened MV leaflets with large vegetations (likely Libman-Sacks endocarditis) extending on the atrial aspect of both anterior and posterior mitral leaflets.

## Discussion

This case describes the potential risk of cardiotoxicity associated with hydroxychloroquine (HCQ) in a 23-year-old female with a history of systemic lupus erythematosus (SLE) presenting with an ischemic embolic stroke likely due to Libman-Sacks endocarditis vegetations, incidentally found to have hypertrophic cardiomyopathy. Although hydroxychloroquine is generally recommended for patients with SLE at any stage and is well-tolerated, it can lead to severe rare side effects, including retinopathy, hypertrophic cardiomyopathy, QTc prolongation, Torsades de Pointes, and ventricular arrhythmias. Current screening measures involve routine eye examinations to screen for retinopathy, but there is limited literature on screening for the cardiotoxic effects of the drug.

Under the current guidelines by the European Alliance of Associations for Rheumatology (EULAR), hydroxychloroquine is recommended for patients with SLE at any stage at a dose of 5 mg/kg real body weight/day, unless there are severe side effects [1]. HCQ decreases the frequency of lupus nephritis flares, helps prolong disease remission, reduces risks of SLE complications, guards against thrombotic incidents, improves lipid and glucose profiles, prevents renal damage, and reduces cardiovascular risk [2]. HCQ functions by downregulating toll-like receptor activity, thereby reducing the innate immune response [3].

The patient in this case, with a history of SLE on HCQ, experienced a stroke with M1 middle cerebral artery occlusion (Figure 1), indicating a likely embolic arterial source. To find the source of the embolus, a transthoracic echocardiogram (TTE) was conducted and revealed severe left ventricular hypertrophy (LVH) with preserved left ventricular ejection fraction (65%) (Figures 2, 3). Considering this finding and knowledge of several case reports noting the rare risk of HCQ-induced hypertrophic cardiomyopathy and its complications, we believe that the medication is the cause of the hypertrophy in this young patient.

The risk of cardiotoxicity by HCQ usually increases with time, cumulative dose, renal dysfunction, female sex, older age, and genetic predisposition - several characteristics present in this patient. It may take years to develop [3]. This 24-year-old patient, diagnosed with SLE at 18 took 200 mg of HCQ/day. Structural features of HCQ-induced cardiotoxicity commonly appear as concentric hypertrophic cardiomyopathy, as it did in this patient, and may include restrictive features and/or conduction defects. HCQ-induced cardiotoxicity is caused by reversible inhibition of lysosomal enzyme activity resulting in the intracellular buildup of lipids and glycogen within cardiomyocytes [3,4]. Histological findings (ex. vacuolated myocytes, intracellular myeloid bodies, and curvilinear bodies) and genetic testing (ex. sarcomere genes, lysosomal storage defects, etc.) can both prove the etiology of HCQ-induced hypertrophic cardiomyopathy, although neither test was done for this patient [5,6].

This patient had no symptoms revealing cardiotoxic complications. However, hypertrophic cardiomyopathy can cause eventual left ventricular outflow obstruction, mitral regurgitation, heart failure, and myocardial ischemia, leading to eventual symptoms of chest pain, fatigue, dyspnea, and syncope [7]. Failure to recognize HCQ-induced hypertrophic cardiomyopathy and to discontinue the drug can lead to reversible heart failure, irreversible heart failure, or death depending on the progression of the disease [7,8].

## Conclusions

Although no formal guidelines exist to monitor HCQ-induced cardiotoxicity in patients with SLE, clinicians need to recognize the risk factors and structural features of the presentation, screen appropriately using ECG, TTE, and/or TEE if indicated, and diagnose accordingly through clinical history, histological examination, and/or genetic testing. Past cases have shown the reversal of cardiomyopathy with the discontinuation of HCQ, which can be a unique therapeutic challenge requiring collaboration between specialists in rheumatology and cardiology. Upon follow-up, this patient is still on hydroxychloroquine. The context of a patient with moderate to severe complications of SLE should be approached individually considering the patient’s complete history and status using risk-benefit analysis.
